# Service Demand Discovery Mechanism for Mobile Social Networks

**DOI:** 10.3390/s16111982

**Published:** 2016-11-23

**Authors:** Dapeng Wu, Junjie Yan, Honggang Wang, Ruyan Wang

**Affiliations:** 1 Information and Communication Engineering, Chongqing University of Posts and Telecommunications, Chongqing 400065, China; d160101007@stu.cqupt.edu.cn (J.Y.); wangry@cqupt.edu.cn (R.W.); 2 Electrical and Computer Engineering, University of Massachusetts Dartmouth, North Dartmouth, MA 02747, USA; hwang1@umassd.edu

**Keywords:** mobile social networks, service demand discovery, social attribute, virtual directory user

## Abstract

In the last few years, the service demand for wireless data over mobile networks has continually been soaring at a rapid pace. Thereinto, in Mobile Social Networks (MSNs), users can discover adjacent users for establishing temporary local connection and thus sharing already downloaded contents with each other to offload the service demand. Due to the partitioned topology, intermittent connection and social feature in such a network, the service demand discovery is challenging. In particular, the service demand discovery is exploited to identify the best relay user through the service registration, service selection and service activation. In order to maximize the utilization of limited network resources, a hybrid service demand discovery architecture, such as a Virtual Dictionary User (VDU) is proposed in this paper. Based on the historical data of movement, users can discover their relationships with others. Subsequently, according to the users activity, VDU is selected to facilitate the service registration procedure. Further, the service information outside of a home community can be obtained through the Global Active User (GAU) to support the service selection. To provide the Quality of Service (QoS), the Service Providing User (SPU) is chosen among multiple candidates. Numerical results show that, when compared with other classical service algorithms, the proposed scheme can improve the successful service demand discovery ratio by 25% under reduced overheads.

## 1. Introduction

To satisfy the rapidly-growing requirements of wireless data services recently, mobile users can also share the demand services during their intermittent encounters for offloading traffic into Mobile Social Networks (MSNs), which are a type of delay-tolerant network with the social relations of users [1,2,3]. However, users are dynamically moving and strongly socially related in MSNs; this has significant research challenges for service demand discovery in such a mobile environment. In order to enhance the Quality of Experience (QoE), designing a service demand discovery mechanism is crucial for users to access the network resources anytime and anywhere. Generally speaking, the service discovery procedure can be divided into three parts: service registration, service selection and service activation. On the one hand, the Service Providing User (SPU) should notify other users of its service information, and then, this service information is inquired and obtained by Service Demanding Users (SDUs). On the other hand, by selecting the proper SPU from several candidates, packets will be forwarded to the SPU to initiate the selected service.

Usually, service demand discovery mechanisms comprise two categories: the centralized and distributed mechanisms. In particular, Virtual Dictionary Users (VDUs) should be determined with a pre-defined method for the centralized mechanisms, in which service information is stored. Subsequently, SDUs send the inquiry information to the VDU to request a service. Finally, the proper SPUs can be chosen to provide demand service; whereas, for distributed mechanisms, the service registration packets and inquiry packets are broadcast across the network. Consequently, the service information can be obtained by all of the users in the network, by which SPUs can be selected to complete the service demand discovery.

Obviously, the service information is maintained by VDUs in centralized mechanisms; thus, the service registration can be achieved by the interaction between SPUs and VDUs. However, the service registration information should be flooded throughout the network in distributed mechanisms. Therefore, the transmission range of service information and the service update consumption are relatively small in centralized mechanisms. However, by checking the locally-maintained service information, distributed mechanisms can achieve the service discovery with fewer inquiry packets. As can be seen, two kinds of mechanisms have different advantages in different service discovery stages. Eventually, the conclusion can be drawn that these two kinds of mechanisms are not directly applicable for MSNs, which means a more suitable service demand discovery mechanism is needed.

Currently, several key issues need to be addressed when designing a service demand discovery mechanism for MSNs. Firstly, based on the actual measurement of the user traces, a particular phenomenon named “Big World, Small World” has been found in MSNs [4,5,6]. Usually, the network topology can be logically divided into communities according to the concentration degree of users. Users move within their home communities with certain probabilities. Meanwhile, users can also roam around other communities. Obviously, the relationship between users of the same community is closer, whereas the inter-community user relationship is relatively loose [7,8,9]. As mentioned above, during the service registration, the diffusion range of service information packets should be constrained. Therefore, the user relationship should be exploited to achieve quick and cost-efficient service information update. Moreover, the maintenance method of service information should also be designed carefully. Secondly, due to the sparse user distribution, the connection between SPU and SDU has discontinuous characteristics and is difficult to evaluate. Besides, multiple SPUs usually respond to the request from the SDU. Obviously, during service selection, the link reliability between SPU and SDU should be guaranteed, which implies that the service ability of each SPU for a given SDU should be evaluated. Thirdly, the packet forwarding procedure between the selected SPU and SDU should be achieved in a cost-efficient manner. According to the network structure based on user relationships, there are two kinds of relationships between the selected SPU and SDU, belonging to the same community and different communities. To control the redundant packet copies, the relay user selections for different relationships should be designed separately [10,11,12,13,14,15,16,17,18,19,20].

In order to minimize the overhead and resource consumption, the social features should be fully utilized for the service demand discovery procedure. Obviously, the user with a high activity degree can encounter more users in a given period, and then, the spreading degree of its carried packets is also large. Based on the social theory, users can be divided into two types: Local Active User (LAU) and Global Active User (GAU) in the network [21], where LAU is more active in its home community, and GAU is relatively more active within the whole network. With the assistance of LAU and GAU, the performance of service demand discovery can be improved effectively.

Combining the advantages between centralized and distributed service demand discovery mechanisms, a hybrid service discovery strategy, namely the Social Attribute-aware Service demand Discovery mechanism (SASD), is proposed in this paper, which fully utilizes the relationship between users. Firstly, users in the network are divided into communities in a distributed manner. Moreover, user activity degrees are estimated, by which GAUs and LAUs are selected. During the service registration, VDUs for each community are chosen based on their relationships and available buffer resources. Thus, the diffusion and update of service registration packets can be restricted into their home community. Subsequently, the service information outside of the home community of an SDU can be provided by the GAU for service selection. To guarantee the service availability, the user with the highest service ability is selected as the SPU. Finally, the service can be activated between the corresponding SDU and SPU.

The main contributions of the paper are summarized as follows:

- A user relationship evaluation scheme for MSNs is introduced. With our defined belongingness degree, the user relationship can be evaluated, and then, users in the network can be divided into communities logically. Additionally, with the constrained label propagation method, our proposed scheme can be achieved under low complexity, thus a large amount of network resources can be saved.
- A user activity degree estimation method is designed. According to the historical information of movement, the local activity degree can be evaluated. Moreover, based on the movement status transition recorded locally, the global activity degree can be obtained in a distributed manner. As a result, the difference of user activities can be distinguished dynamically, which makes the selecting on VDU and relays more reasonable.
- Combining the advantages between distributed and centralized service demand discovery mechanisms, a hybrid service demand discovery architecture is introduced. VDU is selected in each community according to the activity degree and available buffer space, which can effectively restrict the diffusion range of the service information.
- To minimize the overhead, the service registration is designed based on our encounter probability estimation method; thus, the best relay can be selected to forward the packets to the local VDU. With the assistance of the GAU, the service inquiry to other communities can be achieved, and the optimal SPU selection method based on the service ability evaluation results is introduced. Finally, by comparing the meeting frequencies to the destination community of encountered users, the copy number of service packets can be reduced to achieve the service activation.

The remainder of the paper is organized as follows. In Section 2, some related works are surveyed. Communities logically can be divided based on the relationship between users in Section 3. The users’ activity degree estimation methods are proposed in Section 4. In Section 5, based on the results of social attribute evaluation, a hybrid service demand discovery mechanism is carefully designed. In Section 6, we evaluate the effectiveness of the proposed mechanism and compare the proposed mechanism with previous works. Finally, the conclusion and the future works are given in Section 7. The glossary of this paper is shown in Table 1 for easy reading.

## 2. Related Work

Customized for MSNs, several service demand discovery mechanisms are introduced by researchers. A centralized service discovery mechanism is proposed in [22], where the SPU forwards its service information to the VDU according to the shortest distance rule. Further, each VDU spreads its gathered information to other users in the network.

In a centralized manner, the service information is stored in the VDU with the distributed hash table structure [23]. Consequently, only the keyword of service inquiry information is sent from the SDU to the VDU. Thus, the overhead can be constrained.

According to the social relationships of users, the work in [24] utilized an iterative agent method to select some VDUs from all of the users in the network [25]. Further, the inquiry information and the service registration information can be maintained within a limited range.

From the above-mentioned works [22,23,24], the number of VDUs is determined in an off-line manner, and it is not adaptable to the network state. As a result, a large amount of redundant registration information should be maintained by the VDU, so that the scalability of these mechanisms is limited.

By describing the service information with the Bloom filter, the authors in [26] introduced a distributed service discovery mechanism, where the service information is broadcast by SPUs to their neighbors. Finally, all users in the network can obtain service information.

To speed up the response of service discovery, the minimal delay service discovery mechanism is designed in [27]. The encounter interval between users is estimated by SDUs in a distributed manner, and then, the SPU with the minimal delay will respond to the SDU.

Obviously, service information and service inquiry packets are broadcast by the SPU and SDU respectively, in distributed service discovery mechanisms, so network resources will be wasted on flooding these packets.

A context-based service discovery strategy is introduced in [28]. According to the context information, information from multiple SPUs can be obtained by the SDU. However, multiple response information will be sent to the SDU; thus, constraints should be set for the service activation. As a result, the success ratio of service activation can be improved.

To obtain multiple dimensions of user relationships, the residence periods of the SDU and SPU are estimated in [29]. Moreover, a more stable SPU is selected to provide service for SDUs. Obviously, under scenarios with a sparse user distribution, the connection between users is disrupted frequently. Additionally, without considering the social feature, the theoretical mobility pattern is quite different; therefore, the conclusion drawn from the paper needs to be perfected for MSNs.

Based on the clustering characteristics of mobile users, a hybrid service demand discovery strategy is proposed in [30]. With the proposed cluster structure, the service information can be forwarded flexibly. Combining the advantage of centralized and distributed service demand discovery mechanisms, the tradeoff between the maintenance overhead of service status and the transmission flexibility of service information can be achieved. However, without considering the relationship between users, the service activation lacks rationality.

To sum up, multiple features should be taken into consideration while designing the service demand discovery strategy for MSNs, such as the dynamic, buffer and relationships.

## 3. Users Relationship Evaluation

Users are strongly socially related in MSNs. Therefore, making full use of social attributes can effectively improve the success probability of service demand discovery. In particular, a network can be divided into multiple communities logically based on the relationship between users. Traditionally, the relationship between users can be obtained in an off-line manner, but the realization complexity, the discontinuous feature and the distribution feature make it impractical for MSNs. As mentioned above, users are socially correlated, and their relationships gradually stabilize along with the operation of the network. Therefore, according to the weak ties theory, the strength of relationship can be reflected by the number of common neighbors. Different from the traditionally-defined neighbor, neighbors in MSNs are defined by the average encounter times, as shown in Definition 1. Furthermore, the definition on the relationship strength is shown in Definition 2.

**Definition** **1** (Neighbor users)**.** *The user is viewed as a neighbor of user i while their encounter times are higher than the value of $E_{ave}$, where $E_{ave}$ is the average encounter times of user i with other users. Further, all of the users meeting the above constraint constitute the neighbor user set $\Gamma \left(i\right)$ of user i, as shown in Equation (1).*

(1) $$\Gamma \left(i\right)=\left\{v\in V|\left(i,v\right)\in E\right\}$$

**Definition** **2** (Relationship strength)**.** *The Relationship Strength (RS) between user u and v is determined by two parameters, the number of common neighbors and the total neighbors. The first parameter is utilized to illustrate the relationship between users, and the last parameter is utilized to ensure the asymmetric feature of the relationship. the relationship strength from user u to v can be estimated by Equation (2):*

(2) $$b_{u,v}=\frac{\left|\Gamma \left(u\right)\cap \Gamma \left(v\right)\right|}{|\Gamma \left(u\right)|}$$

As mentioned above, users in MSNs form into communities, and their belongingness for each community is quite different. According to the historical information of movement, the belongingness of a user can be determined by its RS with users from the given community, as shown in Equation (3), where $B_{C}\left(u\right)$ is the belongingness of user *u* to community *C*, and $N_{C}\left(u\right)$ denotes the neighbors of user *u* sharing the same community label *C*.

(3) $$B_{C}\left(u\right)=\frac{\sum_{v\in N_{C}\left(u\right)}b_{u,v}}{\left|N_{C}\left(u\right)\right|}$$

Obviously, according to the belongingness, the relationship between users can be evaluated. Because network resources are limited in MSNs, the belongingness status cannot be diffused in the whole scale of the network. Based on the social feature, home communities of neighbor users are always the same, which means the relationship between users and the belongingness for different communities of users can be evaluated according to the information of their neighbors.

To simplify the relationship evaluation, based on the semi-supervised learning method, a label propagation-based relationship evaluation algorithm is proposed in this paper. With our defined constraints, the propagation and update of control information about the belongingness can be restricted.

According to our relationship evaluation algorithm, the label is initialized by users according to their own IP addresses. While users encounter, their encounter times are compared to the average encounter times to identify the neighbor relationship, and then, their belongingness is evaluated according to (2). As can be seen, the overhead of the control information is determined by the label update method [16]. To enhance the robustness of our proposed relationship evaluation algorithm, the asynchronous update method is applied in our paper, as shown in (4); where $L_{\alpha }^{\prime }\left(t\right)$ denotes the candidate label of user *α* at time *t*, $L_{\alpha_{ik}}\left(t-1\right)$ is the label of the neighbor *k* of user *α* in time t−1 and *f* here returns the label occurring with the highest frequency among neighbors; where $\alpha_{i1},\alpha_{i2}\ldots \ldots \alpha_{im}$ are neighbors of user *α*, and their labels have already been updated, whereas $\alpha_{i(m+1)},\ldots \ldots \alpha_{ik}$ are the users whose labels have not been updated.

(4) $$\begin{array}{ll} L_{\alpha }^{\prime }\left(t\right) & =f\left(L_{\alpha_{i1}}\left(t\right),....,L_{\alpha i_{m}}\left(t\right),L_{\alpha_{i\left(m+1\right)}}\left(t-1\right),\right.\\ & \left.....L_{\alpha_{ik}}\left(t-1\right)\right) \end{array}$$

While the candidate label is the same as the label of user *α*, its label remains unchanged. Otherwise, the belongingness of user *α* to these two communities is obtained according to Equation (3). If and only if the belongingness of the candidate label is larger than the belongingness of the original community, the candidate label replaces the original one. Obviously, accurate evaluation of the user relationship can make the estimation of the user activity level accurate and make the design of the service demand discovery mechanism effective.

## 4. Users’ Activity Evaluation

Even though users are in the same community, the service ability of them is also different. In this section, by analyzing the local and global activity degree of each user, the service ability of users can be evaluated, where the local activity degree describes the relationship between directly-related users within the same community, and the global activity degree describes its relationship with the users of other community. As mentioned above, the network can be divided into *k* communities $N=C_{1}\cup C_{2}\cup C_{3}\cdots \cup C_{k}$. For epoch *T*, user *u* records the encounter times with its neighbors, which can be described by $N_{n_{1}}^{u},N_{n_{2}}^{u},N_{n_{3}}^{u}\cdots N_{n_{i}}^{u}$. Accordingly, the average encounter times $N_{ave}^{u}$ between user *u* and its neighbors can be obtained, and the active neighbors can be filtered on the basis of the value $N_{ave}^{u}$, as shown in (5).

(5) $$\Psi_{u}\left(i\right)=\left\{\begin{array}{ll} 1 & N_{n_{i}}^{u}\geq N_{ave}^{u}\\ 0 & N_{n_{i}}^{u}<N_{ave}^{u} \end{array}\right.$$

To distinguish the difference between these active neighbors, all users in the network evaluate their local activity degree $A_{LAU}$ according to the historical movement status information, as shown in Equation (6); where *C* denotes the current community and $\left|C\right|$ denotes the user number of community *C*.

(6) $$A_{LAU}\left(i\right)=\frac{\sum_{u\in C}\Psi_{u}\left(i\right)}{\left|C\right|}$$

Different from the local activity degree, the global activity degree describes the relationship with all users in the network. The user with a higher global activity degree has a closer relationship with users outside or inside of its home community.

As can be seen, the global active users are often roaming around, so the global activity degree $A_{GAU}\left(i\right)$ can be evaluated by Equation (7). Obviously, the higher probability of the GAU roaming around implies that it can provide a wider packet spreading range.

(7) $$A_{GAU}\left(i\right)=\frac{N_{roam}^{i}}{N_{local}^{i}}$$

where $N_{local}^{i}$ is the number of epochs while user *i* stays in its home community and $N_{roam}^{i}$ is the number of epochs while user *i* is roaming. To determine the user status of each epoch, the home communities of all encountered users are maintained locally. While more encountered users are from the same home community, the user status of this epoch is local. Otherwise, the status is roaming.

For period *T*, the proportion of $N_{local}^{i}$ and $N_{roam}^{i}$ is determined by the probability that the user stays in its home community and roams around, as shown in Equation (8); where $\pi_{l}^{\left(i\right)}$ and $\pi_{r}^{\left(i\right)}$ denotes the probability that the user stays in its home community and roams around for epoch *T*.

(8) $$\frac{N_{roam}^{i}}{N_{local}^{i}}=\frac{\pi_{r}^{\left(i\right)}}{\pi_{l}^{\left(i\right)}}$$

As mentioned above, there are two kinds of statuses, local and roaming, respectively. Moreover, the transition between these two kinds of movement statuses can be described as follows.

$P_{l}$ denotes the probability that a user stays in its home community, and it will remain unchanged in the next epoch. $P_{r}$ denotes the probability that the user is roaming around, and it will remain roaming around in the next epoch. Obviously, one status lasts and corresponds with one epoch. According to the status transfer theorem introduced by Markov theory, the stationary equation for $\pi_{l}^{\left(i\right)}$ and $\pi_{r}^{\left(i\right)}$ can be illustrated by Equations (9) and (10).

(9) $$\left(\begin{array}{c} \pi_{l}^{\left(i\right)}\\ \pi_{r}^{\left(i\right)} \end{array}\right)=\left(\pi_{l}^{\left(i\right)}\;\;\;\;\;\;\;\pi_{r}^{\left(i\right)}\right)\times \left(\begin{array}{cc} P_{l} & 1-P_{l}\\ 1-P_{r} & P_{r} \end{array}\right)$$

(10) $$\pi_{l}^{\left(i\right)}\;\;\;+\;\;\;\;\pi_{r}^{\left(i\right)}\;\;\;=1$$

Further, the expression of $\pi_{l}^{\left(i\right)}$ and $\pi_{r}^{\left(i\right)}$ can be obtained as shown in Equations (11) and (12).

(11) $$\pi_{l}^{\left(i\right)}=\frac{1-p_{r}^{\left(i\right)}}{2-p_{l}^{\left(i\right)}-p_{r}^{\left(i\right)}}$$

(12) $$\pi_{r}^{\left(i\right)}=\frac{1-p_{l}^{\left(i\right)}}{2-p_{l}^{\left(i\right)}-p_{r}^{\left(i\right)}}$$

Combining Equation (9) to Equation (12), the value of $A_{GAN}\left(i\right)$ can be estimated, as shown in Equation (13).

(13) $$A_{GAU}\left(i\right)=\frac{1-p_{l}^{\left(i\right)}}{1-p_{r}^{\left(i\right)}}$$

According to the above method, each user in the network can evaluate its own global activity degree in a distributed manner.

## 5. Service Discovery Mechanism

As mentioned previously, neither the distributed, nor the centralized service demand discovery mechanism is suitable for MSNs. Though the spread degree of service information is higher for the distributed mechanism, its overhead is difficult to control. Comparatively speaking, the centralized mechanism limits the overhead by storing service information in the VDU; thus, a large number of information should be handled by the VDU, resulting in a single-point failure problem due to discontinuous connection and energy depletion.

To achieve the tradeoff between the spread degree of service information and the overhead of service discovery, a hybrid service demand discovery mechanism is designed, which consists of the service registration, service inquiry and service activation. Specially, the VDU is introduced to handle the registration and the inquiry of service information. In order to reduce the overhead, VDUs only provide service for users in a finite region. According to the aggregating character of users, each community can be regarded as a unit region.

As can be seen, SPUs register their service information to the corresponding VDU in a distributed manner. With the continuous motion of users, each VDU can obtain the needed service information from other communities in the best effort manner. Moreover, VDUs are in charge of the service inquiring from SDUs in a centralized manner. Subsequently, by selecting the user with higher service ability as the SPU, the reliability of the service between the SDU and SPU can be guaranteed. Finally, the service activation procedure can be completed by selecting the user with the highest encounter frequency to the SPU or roaming to the destination community with higher frequency as the relay.

### 5.1. Service Registration and Cancellation

To achieve ubiquitous computing under the distributed network, SPUs send their service registration information to the VDU of the same home community. Apparently, the selection of the VDU is one of the most important issues in this stage. Considering the user relationship within a community, the user with higher $A_{LAU}\left(i\right)$ will encounter other users more frequently; thus, it can help diffuse service registration information to other users. Meanwhile, due to the dynamic feature in MSNs, the service status changes constantly, and a large amount of information should be maintained by the VDU, so the buffer capacity is the other factor for VDU selection. Therefore, to register service information and to handle service inquiries cost-efficiently, the VDU is selected by jointly considering $A_{LAU}\left(i\right)$ and the residual buffer. According to (14), users can estimate their suitability as VDUs in a distributed manner. Furthermore, by comparing with each other, the selection of the VDU can be accomplished.

(14) $$N_{VDU}=A_{LAU}\left(i\right)\times \frac{B_{i}-B_{i}^{busy}}{B_{i}}$$

where $A_{LAU}\left(i\right)$ denotes the local activity degree of user *i*, $B_{i}$ denotes the buffer capacity of user *i* and $B_{i}^{busy}$ denotes the occupied buffer capacity of user *i*.

**Figure 1:**
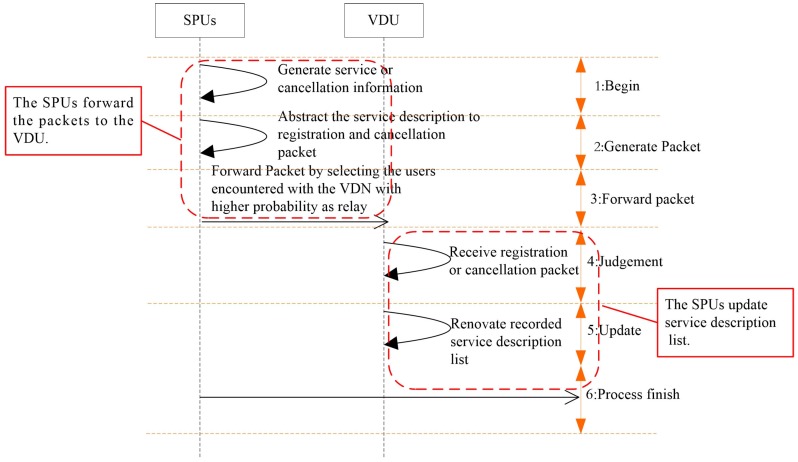
Service registration procedures.

Additionally, when a registered SPU leaves the network or the service information survival time expires, the SPU should send the service cancellation information to the VDU. The process of service registration and cancellation is shown in Figure 1.

After a service is generated by the SPU, the packet containing the service description information, such as the SPU address, service type and survival time is, forwarded to the VDU of the same home community. Subsequently, the VDU stores the received service information until the survival time expires. Additionally, to ensure the validity of services, the service cancellation information should be sent to the VDU when the service is unavailable.

In the service registration process, the distance between the VDU and SPU always contains several hops, and a large amount of resources can be saved by selecting users encountered with the VDU with high probabilities as relays. To limit the diffusion range, a meeting probability-aware packet transmitting method is introduced.

While user *i* and *j* encounter for the first time, the initial value $P_{\mathrm{init}}$ of meeting the probability is assigned, as shown in Equation (15). Simultaneously, the meeting time $t_{1}$ is recorded.

(15) $$P_{1}\left(i,j\right)=P_{\mathrm{init}}$$

While user *i* and *j* encounter again, the meeting time $t_{2}$ is also recorded, and the meeting probability $P_{2}\left(i,j\right)$ is updated, as shown in Equation (16).

(16) $$P_{1}\left(i,j\right)=P_{\mathrm{init}}$$

(17) $$P_{\mathrm{new}}\left(i,j\right)=P_{\mathrm{old}}\times \gamma^{\left(t_{\mathrm{new}}-t_{\mathrm{ave}}\right)}$$

where *γ* is the attenuation factor, $t_{\mathrm{new}}$ denotes the last meeting interval and $t_{\mathrm{ave}}$ denotes the average meeting interval. According to Equation (17), the conclusion can be drawn that the meeting probability will decrease dramatically while the meeting interval between user *i* and *j* becomes larger, namely $t_{\mathrm{new}}\to \infty$, $P_{\mathrm{new}}\left(i,j\right)\to 0$.

According to Equations (15)–(17), the meeting probability can be updated, as shown in Equation (18).

(18) $$P_{\mathrm{avg}(2)}\left(i,j\right)=\frac{\left(P_{1}\left(i,j\right)\times \frac{1}{t_{1}}+P_{2}\left(i,j\right)\times \frac{1}{t_{2}}\right)}{\frac{1}{t_{1}}+\frac{1}{t_{2}}}$$

With the continuous motion of users, the meeting probability between user *i* and *j* will be updated persistently, as shown in Equation (19).

(19) $$\begin{array}{c} P_{\mathrm{avg}(n)}\left(i,j\right)=P_{\mathrm{avg}(n-1)}\left(i,j\right)\times \sum_{m=1}^{n-1}\frac{1}{t_{m}}/\sum_{m=1}^{n}\frac{1}{t_{m}}\\ \;\;\;\;\;\;\;\;\;\;\;\;+P_{n}\left(i,j\right)\times \frac{1}{t_{n}}/\sum_{m=1}^{n}\frac{1}{t_{m}} \end{array}$$

During the service registration, the packet forwarding ability to the VDU of the encountered users is evaluated according to the proposed method; thus, the service registration packet can be successfully delivered to the VDU cost-efficiently. In this way, the probability of service registration can be enhanced.

### 5.2. Service Selection

Obviously, the current service information is maintained by the VDU, and the SDU can obtain the information about the available service according to the inquiry results. Obviously, there are probably several SPUs matching the inquiry. As can be seen, the network performance can be improved by selecting the optimal SPU. Due to the distributed feature, the global network status is not perceivable for the SDU, so the selection of SPUs cannot be accomplished in a cost-efficient manner.

**Figure 2:**
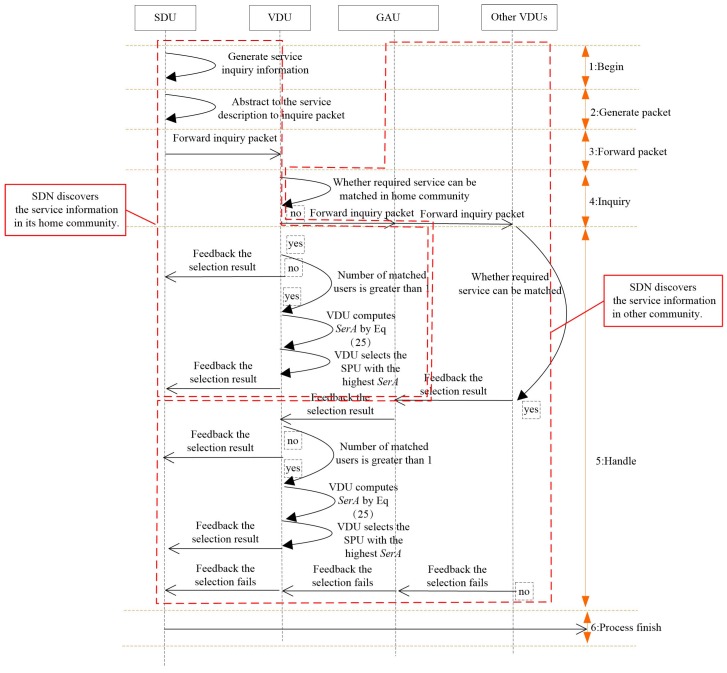
Service selection procedures.

In order to enhance the successful discovery rate, a service ability-aware SPU selection method is introduced. By selecting the user with the highest service ability as the SPU, the results of service inquiry can be replied to more quickly, and the relay times can also be reduced. The principle of the proposed service selection method is shown in Figure 2.

After receiving the service inquiry packet from the SDU, the VDU searches the related information within its buffer. If the inquired service can be matched by the SPU of the same community, the SPU with the highest service ability is selected to provide the service for the SDU. Exceptionally, service status outside the community of the SDU is often requested. To reduce the resource consumption in this process, the global activity degrees $A_{GAU}\left(i\right)$ of all users of a given community are compared by the VDU, and the user with the highest global activity degree $\max\{A_{GAU}\left(i_{1}\right),A_{GAU}\left(i_{2}\right),\cdots ,A_{GAU}\left(i_{n}\right)\}$ is selected to relay service inquiry packets to VDUs of other communities. Subsequently, the service information is checked by VDUs after receiving the service inquiry packets from the GAU in a distributed manner, and then, the inquired results are replied back to the GAU. Until the GAU encounters the VDU matching the inquired SDU, by repeating the above-mentioned local SPU selection procedure, the SPU with the highest service ability is selected for the inquiring SDU.

Obviously, the service ability evaluation is the most important issue for the service selection stage, where the connection strength and service rate are two key factors. Respectively, the connection duration and connection times can be reflected by the connection strength, and the number of forwarded packets can be determined by the service rate. As a result, the higher service ability a user has, the larger the number of packets it can forward for other users.

For a given user *i*, the duration of n−th connection is determined by the connection establishing time $t_{up}\left(n\right)$ and the link disconnection time $t_{down}\left(n\right)$, as shown in Equation (20).

(20) $$t_{up\_state}^{i}\left(n\right)=t_{down}^{i}\left(n\right)-t_{up}^{i}\left(n\right)$$

Further, according to the recorded information about the two parameters of each connection, the total connection duration during given period T for user *i*, $sum_{up\_state}^{i}$ can be obtained, as shown in Equation (21).

(21) $$\begin{array}{c} sum_{up\_state}^{i}=\sum_{n=1}^{m}t_{up\_state}^{i}\left(n\right)\\ \;\;\;\;\;\;\;\;\;\;\;=\sum_{n=1}^{m}t_{down}^{i}\left(n\right)-t_{up}^{i}\left(n\right) \end{array}$$

Obviously, in given period *T*, the connection strength is related to the connection duration directly, as shown in Equation (22).

(22) $$\lambda_{i}=sum_{up\_state}^{i}/T$$

Additionally, the service rate is determined by the number of forwarded packets under a given connection, as shown in Equation (23), where $t_{up\_state}^{i}\left(n\right)$ denotes the duration of the n−th connection, $a_{n}$ denotes the number of forwarded packets and $N_{i}$ denotes the number of connections.

(23) $${\bar{\mu }}_{i}=\sum_{n=1}^{N_{i}}\mu_{i}\left(n\right)/N_{i}=\sum_{n=1}^{N_{i}}\frac{a_{i}\left(n\right)}{t_{up\_state}^{i}\left(n\right)}/N_{i}$$

Combining the connection strength and service rate, the service ability of user *i*, $S\mathrm{er}A_{i}$ can be evaluated accurately, as shown in Equation (24).

(24) $$S\mathrm{er}A_{i}=\lambda_{i}\cdot {\bar{\mu }}_{i}$$

According to the above method, the service inquiry packet can be forwarded to other communities more quickly with the assistance of the GAU. Based on the interactions between the GAU and VDUs, the information about SPUs can be obtained by the corresponding VDU. To further restrict the overhead of the service selection, the transmission delay is utilized to filter the feedback packets from the GAU. After the VDU receives the feedback packets, a timer is triggered, and the service ability for each SPU is compared after the timer expires. Thus, the service selection can be accomplished.

### 5.3. Service Activation

To exploit the user relationship, the home community of an SPU is checked firstly after the inquiry results are replied to by the VDU. Further, the service activation procedure is executed according to the relationship between the SPU and SDU.

**Figure 3:**
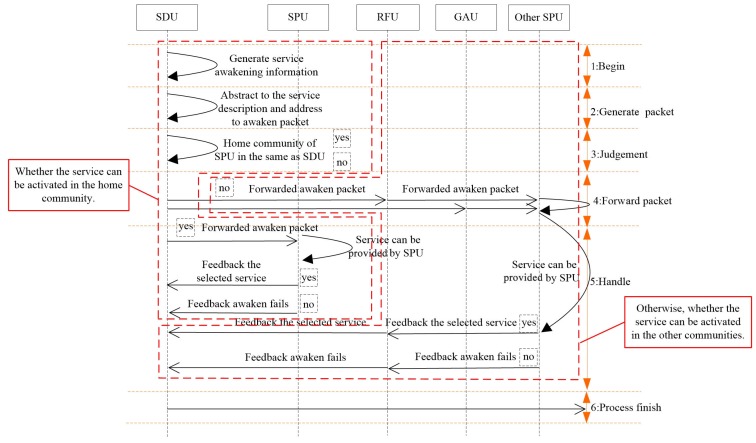
Service activation procedures.

While the SDU and SPU belong to the same community, the SDU forwards the service activation information to the SPU. Generally, packets are forwarded by several relay users in MSNs, which is also applied to the transmission of service activation packets. The principle of the proposed service activation procedures is shown in Figure 3.

Obviously, the meeting interval can be utilized to evaluate the forwarding delay, and the service activation information can be forwarded quickly by selecting the user with the smallest meeting interval to the SPU as the relay. However, due to the dynamic feature, each interval between adjacent connections varies and probably has a large deviation with the historical meeting interval. Therefore, it is unreasonable to estimate the meeting interval with the mean value method. To improve the accuracy of estimating the residual time to the next connection, the average residual time to the next connection between user *i* and *j* is firstly evaluated in Equation (25). According to the meeting interval between the user carrying the service activation packet and its destination, the connection intervals larger than the elapsed time from the last connection are selected as the sample, then those samples are averaged to enhance the estimation accuracy.

(25) $${\bar{T}}_{i}\left(j|t^{\prime }\right)=\sum_{s=1}^{k}f_{i}^{s}\left(j\right)/\left|\{t_{i}\left(j\right)>t^{\prime }\}\right|$$

(26) $$f_{i}^{s}\left(j\right)=\left\{\begin{array}{ll} t_{i}\left(j\right)-t^{\prime } & if\;t_{i}\left(j\right)>t^{\prime }\\ 0 & else \end{array}\right.$$

where ${\bar{T}}_{i}\left(j|t^{\prime }\right)$ denotes the average residual time to the next connection between user *i* and *j*, *K* denotes the connection times and $t_{i}\left(j\right)$ denotes the connection interval.

Apparently, the deviation degree between the sample value and mean values can be denoted by the variance. Further, the supremum of residual time to the next connection can be determined by the standard deviation, as shown in Equation (27). Consequently, the meeting interval between the SPU and SDU can be evaluated accurately.

(27) $$TM_{i}\left(j|t^{\prime }\right)={\bar{T}}_{i}\left(j|t^{\prime }\right)+\sqrt{\frac{1}{k}\sum_{s=1}^{k}{\left({\bar{T}}_{i}\left(j|t^{\prime }\right)-f_{i}^{s}\right)}^{2}}$$

Comparatively speaking, while the SPU and SDU belong to different communities, the relay user selection should consider the forwarding cost. According to the social theory, the community scale varies. Therefore, except the roaming times, the roaming probability to the destination community is also utilized to evaluate the relationship between a given user and its destination community, and the evaluation method is shown in Equation (28).

(28) $$A_{C(d)}^{i}\left(t\right)=N_{C(d)}^{i}\left(t\right)/\sum_{C_{roam}\in R}N_{C_{roam}}^{i}\left(t\right)$$

where $N_{C(d)}^{i}\left(t\right)$ and $N_{C_{roam}}^{i}\left(t\right)$ denote the roaming times of user *i* to the destination community and to community $C_{roam}$, respectively, and *R* denotes the community set to which user *i* has been. If $A_{C(d)}^{j}\left(t\right)>A_{C(d)}^{i}\left(t\right)$, the Roaming to destination community Frequency User (RFU) *j* will help carry and forward the service activation packet. Consequently, the service activation packet can reach its destination community, and then, the above-mentioned activation process will be executed. In case there are no users roaming to the destination community, the user with the highest global activity degree $A_{GAU}{\left(i\right)}_{\max}$ is selected to forward the service activation packet to the SPU. The algorithmic pseudocode of our proposed SASD is shown in Algorithm 1.

**Algorithm 1** Social attribute-aware service demand discovery mechanism.**Initialization:** Community division according to Part III; 1:  Calculating the local activity degree $A_{LAU}$ and the global activity degree $A_{GAU}$ for each user according to the Equations (6) and (13); 2:  When a service is generated by the SPU; 3:  Its service description information, such as the SPU address, service type and survival time, is forwarded to the VDU, which is selected by the maximum local activity degree $A_{LAU}$; 4:  When a service is needed by the SDU; 5:  The SDU will send the service inquiry packet to the VDU, and the VDU searches the related information within its buffer and then matches the SPUs to the SDU. 6:  **if** The SDU and SPUs are in the same community **then** 7:   The SPU will provide the service for the SDU; 8:  **else** 9:   **if** The SDU and SPUs are not in the same community **then**10:    The SDU chooses the maximum global activity degree $A_{GAU}$ user who will find the other VDUs to provide service;11:   **end if**12:  **end if**13:  END

## 6. Numerical Results

### 6.1. Parameter Setting

To compare the performance of our proposed SASD with those of other popular service demand discovery mechanisms, we establish a simulation environment using ONE (Opportunistic Network Environment) [31], which is a powerful framework for generating different movement models, running the simulation with various protocols, visualizing simulations in real time and outputting/post-processing the results.

In the simulation, we compare SASD with DSDM (Distributed Service Discovery Model) [22] and DMBSLP (Discovery Mechanism Based on Layer Protocol) [32]. DSDM is a typical hybrid service demand discovery mechanism, and multiple VDUs are selected according to distances between VDUs and SPUs, whereas DMBSLP is a typical distributed mechanism, where users have identical status.

The impacts of the service request interval on different performance metrics is evaluated in the simulation. In particular, a map-based user mobility model is utilized to evaluate the service demand discovery mechanisms. This model restricts the movements of users to actual streets in an imported map, which is a 4500 m × 3400 m-sized map of Helsinki, Finland. Moreover, the transmission ranges are assumed to be 10 m. The transmission rate for all users is 250 KBps, whereas the buffer size is 20 MB. The users move at a speed within 1–3 m/s. The packet size is normally distributed with an average size of 1 MB.

Along with the change of service request interval, the performances of SASD, DSDM and DMBSLP are compared. Moreover, four metrics are simulated, and they are SRSProb (Service Register Success Probability), Service Query Success Probability (SQSProb), Service Discovery Success Probability (SDSProb) and Overhead Ratio (OR) respectively.

### 6.2. Complexity Analysis of the User Relationship Evaluation Method

As mentioned before, the user relationship evaluation should be achieved in a cost-efficient manner. Obviously, the control overhead is determined by the complexity of the user relationship evaluation method, and the time complexity analysis for the user relationship evaluation method is analyzed in detail in this sub-section. The overhead of our proposed method mainly consists of two parts, the overhead during the initialization stage and during the label update stage.

Assume the network *N* has $n_{N}$ users, and the encounter times between users is *m*. With our proposed relationship evaluation method, the network can be divided into *k* communities, as $N=C_{1}\cup C_{2}\cup C_{3}\cdots \cup C_{k}$; moreover, community $C_{i}$ consists of several users, namely $C_{i}=\left\{h_{1},\;\;\;\;\;\;\;h_{2},\;\;\;\;\;\;h_{3},\;\;\;\;\;\ldots \right\}$.

There are two constraints that should be satisfied in our method:

(1) $\forall C_{i}$, $C_{j}\in N$, $C_{j}\in N$ and i≠j.
(2) $N=C_{1}\cup C_{2}\cup C_{3}\cdots \cup C_{k}$ and $n_{N}=\;\;\;|C_{1}\;\;|\;+\;|\;C_{2}\;|\;+\;\;\;\ldots \;\;\;+\;|C_{k}\;\;|\;$, where $n_{C_{i}}=\;\;\;\left|C_{i}\;\;\right|$.

In the initialization stage of user relationship evaluation, users in the network use their unique IDs as community labels, and the time complexity is $O(n_{N})$. Obviously, this process is realized in a distributed manner, so the overhead is ignorable.

Technically, the status information is updated after users encounter. Therefore, the time complexity of the label updating stage is related to the user encounter interval and user encounter times. While user *i* has $N_{i}$ $N_{i}\in \left(0,m\right)$ neighbors in the community, the community label of user *i* is updated according to its neighbor status. Obviously, from Algorithm 1, the complexity of our method is O(N). The conclusion can be drawn that the time complexity of the proposed relationship evaluation method is nearly the linear complexity.

### 6.3. Network Performance under Different Service Request Intervals

**Figure 4:**
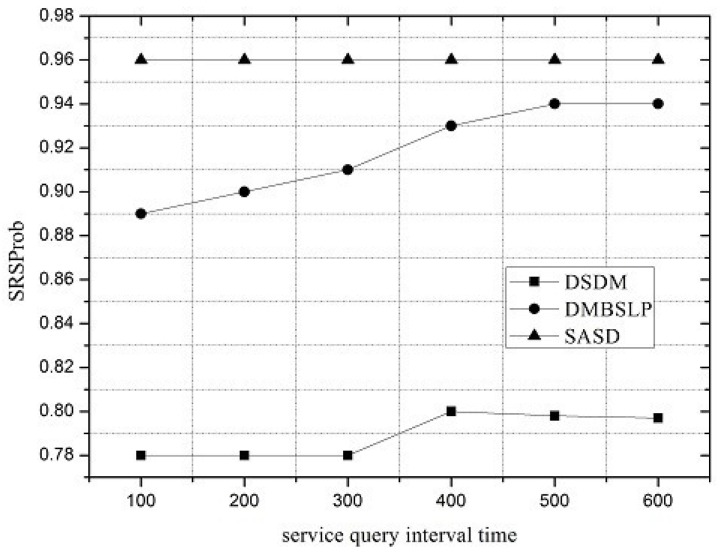
Service Register Success Probability (SRSProb). SASD, Social Attribute-aware Service demand Discovery mechanism.

**Figure 5:**
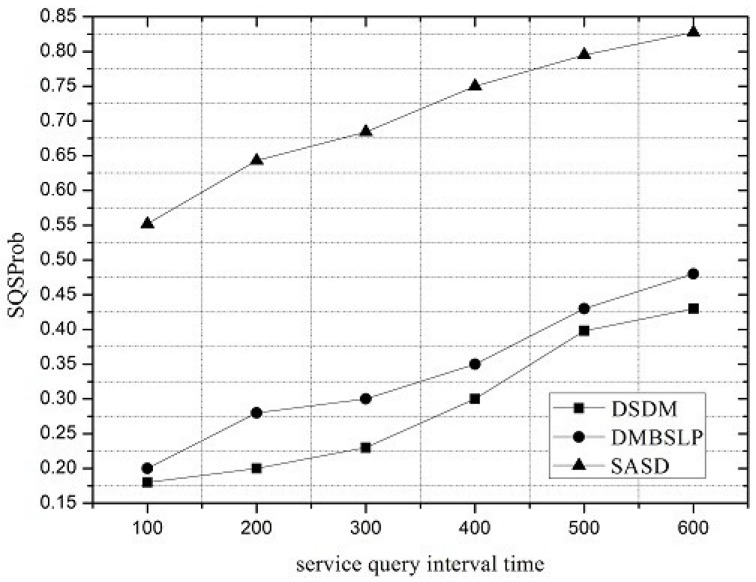
Service Query Success Probability (SQSProb).

**Figure 6:**
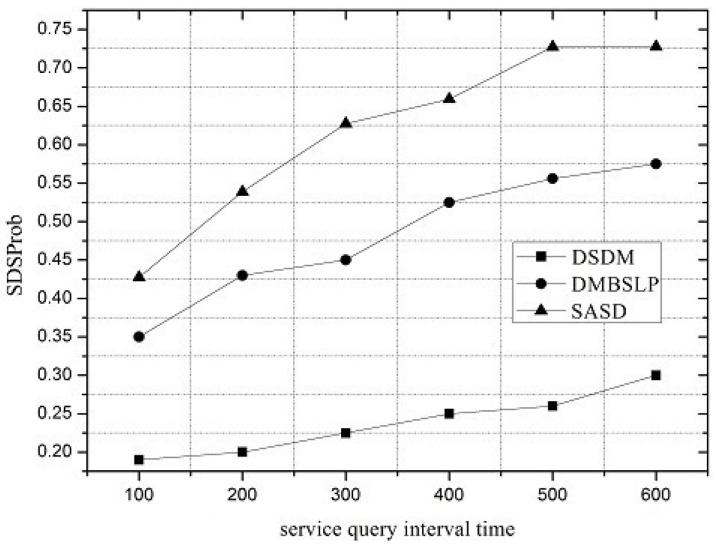
Service Discovery Success Probability (SDSProb).

**Figure 7:**
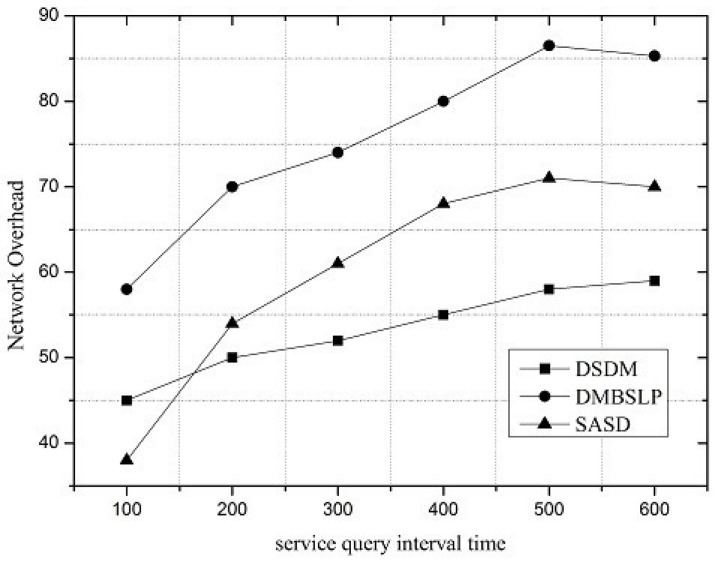
Overhead ratio.

Under different network loads, the performance of the service demand discovery mechanism varies. The service request interval can be utilized to reflect the network load. SRSProb, SQSProb, SDSProb and OR are shown in Figure 4, Figure 5, Figure 6 and Figure 7. On the whole, the SASD combines the advantages between centralized and distributed service demand discovery mechanisms. What is more, the SASD also unites the social attribute between users for service demand discovery. They all make our proposed mechanism better than other two mechanisms.

Figure 4 shows that the SRSProb of SASD and DSDM keeps unchanged under different service request intervals, which is higher than those of DSDM and DMBSLP. Under the distributed packet transmitting method, the limited buffer resources of the user should be utilized reasonably. While the service request interval increases, less service request packets will be injected into the network; thus, the residual buffer can be reserved for the service registration from other users.

From results shown in Figure 5, the SQSProbs for all three mechanisms increase with the interval of service request packets. While the interval of service request becomes larger, the number of service query packets can be reduced; thus, the delivery ratio to the VDU increases because of the mitigated resource contention. Compared with DSDM and DMBSLP, the SQSProb of SASD is much higher.

As illustrated in Figure 6, the SDSProb of SASD is much higher than that of DSDM and 28.5% higher than that of DMBSLP. According to SRSProb and SQSProb, the advantage of SASD is obvious.

Additionally, the overhead ratio is compared in Figure 7. In the distributed method, packets will be relayed and forwarded many times by DMBSLP; therefore, its overhead ratio is higher than those of the other two mechanisms. With the VDN introduced into the network, the diffusion range of service status update packets is limited, so the overhead ratios of SASD and DSDM are quite lower than DMBSLP, but because of the service discovery architecture, the overhead rate of SASD is 13.4% higher than that of DMBSLP.

## 7. Conclusions

To improve the performance of the service demand discovery mechanism in MSNs, a social attribute-aware service demand discovery mechanism is proposed in this paper. Based on the relationship between users, a network can be divided into communities logically. Further, the LAU and GAU are evaluated according to the information obtained from the historical user movement. Compared with the existing DSDM and DMBSLP, SASD can significantly improve the success probability of service discovery and reduce service discovery overhead. In addition, our future work is as follows:

According to the network structure detection results, only one VDU is selected for each community in our paper. When the community is large, the performance of service discovery may decline. We should adaptively adjust the number of VDUs according to the size of each community. Meanwhile, VDUs may be invalid because of energy depletion, so it is necessary to design a service discovery strategy for multi-VDU cooperation in each community. As to the hybrid service discovery, multiple parameters should be considered to select VDUs, such as energy and bandwidth of users, which makes the selected VDUs more practical.

## Figures and Tables

**Table 1:** The glossary of this paper.

Notation	Definition
MSNs	Mobile Social Networks
VDU	Virtual Dictionary User
LAU	Local Active User
GAU	Global Active User
QoS	Quality of Service
QoE	Quality of Experience
SPU	Service Providing User
SDU	Service Demanding User

## References

[1] Wang X., Chen M., Han Z., Wu D.O., Kwon T.T. TOSS: Traffic Offloading by Social Network Service-Based Opportunistic Sharing in Mobile Social Networks. Proceedings of the IEEE INFOCOM.

[2] Ioannidis S., Chaintreau A., Massoulie L. Optimal and Scalable Distribution of Content Updates over a Mobile Social Network. Proceedings of the IEEE INFOCOM.

[3] Wu D.P., Zhang P.N., Wang H.G., Wang C.G., Wang R.Y. (2016). Node Service Ability aware Packet Forwarding Mechanism in Intermittently Connected Wireless Networks. IEEE Trans. Wirel. Commun..

[4] Daly E.M., Haahr M. (2010). The Challenges of Disconnected Delay-Tolerant MANETs. Ad Hoc Netw..

[5] Eyuphan B., Boleslaw K. (2012). Exploiting Friendship Relations for Efficient Routing in Mobile Social Networks. IEEE Trans. Parallel Distrib. Syst..

[6] Sabrina G., Elena P., Gian P.R. (2011). Strangers Help Friends to Communicate in Opportunistic Networks. Comput. Netw..

[7] Wu D.P., Zhang H.P., Wang H.G., Wang C.G., Wang R.Y., Xie Y. (2015). Quality of Protection (QoP)-Driven Data Forwarding for Intermittently Connected Wireless Networks. IEEE Wirel. Commun..

[8] Wang R.Y., Yang H.P., Wang H.G., Wu D.P. (2016). Social Overlapping Community Aware Neighbor Discovery for D2D Communications. IEEE Wirel. Commun..

[9] Wu D.P., Wang Y.Y., Wang H.G., Yang B.R., Wang C.G., Wang R.Y. (2016). Dynamic Coding Control in Social Intermittent Connectivity Wireless Networks. IEEE Trans. Veh. Technol..

[10] Luo C., Min G., Yu F.R., Chen M., Yang L.T., Leung V.C.M. (2013). Energy-Efficient Distributed Relay and Power Control in Cognitive Radio Cooperative Communications. IEEE J. Sel. Areas Commun..

[11] Wu D.P., He J., Wang H.G., Wang C.G., Wang R.Y. (2015). A Hierarchical Packet Forwarding Mechanism for Energy Harvesting Wireless Sensor Networks. IEEE Commun. Mag..

[12] Li Y., Liao C., Wang Y., Wang C.G. (2015). Energy-Efficient Optimal RelaySelection in Cooperative Cellular Networks Based on Double Auction. IEEE Trans. Wirel. Commun..

[13] Peng M., Li Y., Jiang J., Li J., Wang C. (2014). Heterogeneous Cloud Radio Access Networks: A New Perspective for Enhancing spectral and Energy Efficiencies. IEEE Wirel. Commun..

[14] Wu D.P., Yang B.R., Wang H.G., Wang C.G., Wang R.Y. (2016). Privacy-preserving Multimedia Big Data Aggregation in Large-scale Wireless Sensor Networks. ACM Trans. Multimed. Comput. Commun. Appl..

[15] Zhang Z.F., Li Y.X., Yang J. (2014). Energy Efficiency Based on Joint Mobile Node Grouping and Data Packet Fragmentation in Shortrange Communication System. Int. J. Commun. Syst..

[16] Wu D.P., Yang B.R., Wang H.G., Wu D.L., Wang R.Y. (2016). Energy-efficient Data Forwarding Strategy for Heterogeneous WBANs. IEEE Access.

[17] Luo C., Guo S., Guo S., Yang L.T., Min G., Xie X. (2014). Green Communication in Energy Renewable Wireless Mesh Networks: Routing, Rate Control, and Power Allocation. IEEE Trans. Parallel Distrib. Syst..

[18] Lei H., Gao C., Ansari I.S., Guo Y., Zou Y., Pan G., Qaraqe K. (2016). Secrecy outage performance of transmit antenna selection for MIMO underlay cognitive radio systems over Nakagami-m channels. IEEE Trans. Veh. Technol..

[19] Lei H., Zhang H., Ansari I.S., Gao C., Guo Y., Pan G., Qaraqe K. (2016). Secrecy outage performance for SIMO underlay cognitive radio systems with generalized selection combining over Nakagami-m channels. IEEE Trans. Veh. Technol..

[20] Lei H., Gao C., Ansari I.S., Guo Y., Pan G., Qaraqe K. (2016). On physical layer security over SIMO generalized-K fading channels. IEEE Trans. Veh. Technol..

[21] Hui P., Crowcroft J., Yoneki E. (2011). BUBBLE Rap: Social-Based Forwarding in Delay-Tolerant Networks. IEEE Trans. Mob. Comput..

[22] Artail H., Mershad K.W., Hamze H. (2008). DSDM: A Distributed Service Discovery Model for MANETs. IEEE Trans. Parallel Distrib. Syst..

[23] He Q., Yan J., Yang Y. (2013). A Decentralized Service Discovery Approach on Peer-to-Peer Networks. IEEE Trans. Serv. Comput..

[24] Raychoudhury V., Cao J., Wu W. (2011). K-directory Community: Reliable Service Discovery in MANET. Pervasive Mob. Comput..

[25] Khatibi S., Rohani R. Quorum-based Neighbor Discovery in Self-organized Cognitive MANET. Proceedings of the IEEE PIMRC.

[26] Outay F., Veque V., Bouallegue R. Application Layer Versus Cross-layer service Discovery Protocols in MANETs. Proceedings of the IEEE ANTS.

[27] Pasarella A., Kumar M., Conti M. (2011). Minimum-Delay Service Provisioning in Opportunistic Networks. IEEE Trans. Parallel Distrib. Syst..

[28] Maheo Y., Said R. Service Invocation over Content-Based Communication in Disconnected Mobile Ad Hoc Networks. Proceedings of the IEEE AINA.

[29] Wang J.P. (2011). Exploiting Mobility Prediction for Dependable Service Composition in Wireless Mobile Ad Hoc Networks. IEEE Trans. Serv. Comput..

[30] Shim Y.S., Kim Y.S., Lee K.H. A Mobility-based Clustering and Discovery of Web Services in Mobile Ad-hoc Networks. Proceedings of the IEEE ICWS.

[31] Keranen A., Ott J., Karkkainen T. The ONE Simulator for DTN Protocol Evaluation. Proceedings of the ICST.

[32] Wang Z., Bulut E., Boleslaw S.K. Service Discovery for Delay Tolerant Networks. Proceedings of the IEEE GLOBECOM.

